# Deep Sequencing and Bioinformatic Analysis of Lesioned Sciatic Nerves after Crush Injury

**DOI:** 10.1371/journal.pone.0143491

**Published:** 2015-12-02

**Authors:** Sheng Yi, Honghong Zhang, Leilei Gong, Jiancheng Wu, Guangbin Zha, Songlin Zhou, Xiaosong Gu, Bin Yu

**Affiliations:** Jiangsu Key Laboratory of Neuroregeneration, Co-innovation Center of Neuroregeneration, Nantong University, Nantong, Jiangsu, China; University of Szeged, HUNGARY

## Abstract

The peripheral nerve system has an intrinsic regenerative capacity in response to traumatic injury. To better understand the molecular events occurring after peripheral nerve injury, in the current study, a rat model of sciatic nerve crush injury was used. Injured nerves harvested at 0, 1, 4, 7, and 14 days post injury were subjected to deep RNA sequencing for examining global gene expression changes. According to the temporally differential expression patterns of a huge number of genes, 3 distinct phases were defined within the post-injury period of 14 days: the acute, sub-acute, and post-acute stages. Each stage showed its own characteristics of gene expression, which were associated with different categories of diseases and biological functions and canonical pathways. Ingenuity pathway analysis revealed that genes involved in inflammation and immune response were significantly up-regulated in the acute phase, and genes involved in cellular movement, development, and morphology were up-regulated in the sub-acute stage, while the up-regulated genes in the post-acute phase were mainly involved in lipid metabolism, cytoskeleton reorganization, and nerve regeneration. All the data obtained in the current study may help to elucidate the molecular mechanisms underlying peripheral nerve regeneration from the perspective of gene regulation, and to identify potential therapeutic targets for the treatment of peripheral nerve injury.

## Introduction

Peripheral nerve injury is commonly caused by penetrating wound, crush, stretch, ischemia, and other traumatic lesions [1]. The adult peripheral nervous system, unlike the central nervous system, has an intrinsic ability to regenerate after injury. For severe peripheral nerve injuries with substantial defects formed between the proximal and distal nerve stumps, however, fully functional recovery is almost unlikely. Instead, severe nerve injures may cause life-long disability and affect the quality of life in patients.

Peripheral nerve injury elicits a series of complex molecular and cellular events at multiple sites, including the neural cell body, the lesioned portion, the proximal and distal stumps, and the target organ. After peripheral nerve injury, a series of changes, such as axon disruption and Wallerian degeneration will occur, the macrophages and monocytes migrate to remove the axon and myelin debris, while Schwann cells proliferate to form the bands of Bungner, and produce neurotrophic factors and extracellular matrix molecules to stimulate axon regrowth [2–8]. Despite the knowledge of such morphological changes, the molecular mechanisms underlying peripheral nerve injury and regeneration have not yet been fully elucidated. A better understanding of the nerve regenerative process from the molecular aspect may help to identify key elements and pathways, therefore leading to the discovery of new therapeutic targets.

As the longest nerve in the body, the sciatic nerve comprises both motor and sensory fibers, and is widely used for studying peripheral nerve-related physiopathology [9]. The sciatic nerve crush model is one of the most common models of peripheral nerve injury [9, 10]. Similar to other models of sciatic nerve injury (for example, sciatic nerve transection), nerve crush also causes disruption of neuronal axons, but leaves the basal laminae of Schwann cells intact, thus benefiting the regeneration of injured nerves [11]. In the current study, rats were used as the experimental animals because of the similarity in the distribution of peripheral nerve trunks between rats and humans [12], and rats underwent surgery to induce sciatic nerve crush.

Recently, microarray techniques have been widely used to examine the expression changes of thousands of genes and proteins during peripheral nerve regeneration, and a series of key elements, pathways, and biological processes have been identified [13–15]. The obtained findings largely expand the understanding of molecular mechanisms that are responsible for peripheral nerve regeneration. However, cDNA microarray and gene chips have several limitations, such as the reliability and accuracy of measurements, especially for those transcripts with low abundance [16, 17]. The newly-developed deep RNA sequencing represents a high-throughput sequencing technique to directly sequence complementary DNAs, followed by mapping to the genome and obtaining outcomes with a high signal-to-noise ratio, large dynamic range, and high reproducibility [17]. We have previously used deep sequencing to characterize microRNAs changes in dorsal root ganglion (DRG) tissues and nerve stumps after sciatic nerve resection, and identified numerous differentially expressed microRNAs, thus providing a preliminary understanding of microRNA regulation of peripheral nerve injury [18]. In the current study, deep sequencing was used to investigate the gene expression on a genome-wide range in the injured sciatic nerve after sciatic nerve crush. Then, the massive data from deep sequencing were analyzed using ingenuity pathway analysis (IPA) in an attempt to search for key molecules and signaling pathways during peripheral nerve regeneration.

In the current study, we obtained a comprehensive transcriptome sequencing data in the injured sciatic nerve of rats at different time points (0, 1, 4, 7, and 14 days post sciatic nerve crush), and screened out a number of differentially expressed genes at different time points. We, for the first time, performed bioinformatic analysis of top enriched diseases and functions and canonical pathways, with a special focus on four canonical pathways: acute phase response signaling, triggering receptor expressed on myeloid cells (TREM1) signaling, LXR/RXR activation, and axonal guidance signaling, which were highly enriched post sciatic nerve crush. Our results illustrate the dynamic gene changes and the related signaling pathways after peripheral nerve crush injury, thus benefiting the understanding of peripheral nerve regeneration at the molecular level.

## Materials and Methods

### Animal surgery and tissue preparation

All animal procedures were performed in accordance with Institutional Animal Care guideline of Nantong University and ethically approved by the Administration Committee of Experimental Animals, Jiangsu Province, China.

A total of 75 adult, male Sprague-Dawley (SD) rats, weighting 180–220 g, were obtained from the Experimental Animal Center of Nantong University. The animals were randomly divided into 5 groups according to different time points of observation. After animals were anaesthetized by an injection of mixed narcotics (85 mg/kg trichloroacetaldehyde monohydrate, 42 mg/kg magnesium sulfate, and 17 mg/kg sodium pentobarbital), a skin incision was made on the lateral aspect of the mid-thigh of the left hind limb. Then, the sciatic nerve at 10 mm above the bifurcation into the tibial and common fibular nerves was crushed with a forceps at a force of 54 N for 3 times (a period of 10 s for each time). The muscle and skin were then sutured. After surgery, SD rats were housed in temperature- and humidity-controlled large cages with sawdust bedding and allowed free access to water and food. The animals in 5 groups were killed by decapitation at 0, 1, 4, 7, and 14 days after surgery, and sciatic nerve segments of 5 mm in length at the crush site were harvested and stored at –80°C. The rats in the 0 day group underwent sham-surgery on the left sciatic nerve and then killed for harvesting nerve samples.

### RNA and protein extraction

Total RNA was extracted using Trizol (Life technologies, Carlsbed, CA) according to manufacturer’s instructions. Contaminating DNA was removed using RNeasy spin columns (Qiagen, Valencia, CA). The quality of isolated RNA samples was evaluated with an Agilent Bioanalyzer 2100 (Agilent technologies, Santa Clara, CA), while the quantity of RNA samples was determined using a NanoDrop ND-1000 spectrophotometer (Infinigen Biotechnology Inc., City of Industry, CA).

Protein lysates were extracted from lesioned sciatic nerve tissues via direct homogenization, and lysis in a Laemmli sample buffer containing 2% SDS, Tris-HCl (52.5 mM), and protein inhibitors. Protein concentration was determined by the Micro BCA Protein Assay Kit (Pierce, Rockford, IL).

### Deep sequencing

Deep sequencing was performed on the RNA samples. Magnetic beads with Oligo (dT) were used to isolate mRNA from total RNA. Purified mRNA was then mixed with the fragmentation buffer and fragmented into short fragments. Random hexamer-primers were used to synthesize cDNA using these short mRNA fragments as templates. Short double-stranded cDNA fragments were purified, resolved with EB buffer for end reparation and the addition of single nucleotide adenine, and then connected with Illumina sequencing adaptors. Size-suitable fragments were selected and purified by agarose gel electrophoresis and then went through the PCR amplification. The amplified library was sequenced using Illumina HiSeq^TM^2000.

Primary sequencing data (raw reads) was subjected to quality control (QC) to filter out dirty raw reads (raw reads connected with sequence adaptors, reads that contains a > 10% N ratio, and low-quality reads). Clean reads were aligned to the reference sequences with SOAPaligner/SOAP2. Alignment results that passed QC were subjected to downstream analysis (S1 Fig). Overall, high-class transcriptome sequencing data were obtained on the basis of balanced base composition of raw reads (S2A Fig), high quality distribution of bases along reads (S2B Fig) and even distribution of reads on reference genes (S2C Fig).

### Analysis of transcriptomic gene expression

The expression levels of mapped genes were calculated using Reads per kilobase transcriptome per million mapped reads (*RPKM*) method to normalize gene expression levels and to eliminate the influence of the difference of gene length as well as sequencing discrepancy on the calculation of gene expression. Briefly, *RPKM* value of certain gene was calculated with the following formula: RPKM = (10^9^×C)/(N×L), where C is the number of reads that are uniquely aligned to a certain gene, N is the total number of reads that are uniquely aligned to all genes, and L is the number of bases on that certain gene.

Differentially expressed genes were screened for different time points. Genes having a false discover rate (FDR) ≤ 0.001 and a fold change > 2 are considered as differentially expressed.

Bioinformatic analysis was performed with IPA for differentially expressed genes at 1, 4, 7, and 14 days post sciatic nerve crush. A data set of differentially expressed genes containing gene identifiers (Entrez gene ID), corresponding expression values (log_2_Ratio), and P-values was uploaded for core analysis. Diseases and functions and canonical signaling pathways related to differentially expressed genes were analyzed according to the Ingenuity Pathways Knowledge Base (IPKB).

### Quantitative real time qPCR

The same RNA samples as described in RNA extraction and transcriptome sequencing were subjected to real time qPCR to validate the results of transcriptome sequencing. The individual RNA samples (0.5 μg) were used as a template and reverse transcribed to cDNA using the Prime-Script reagent Kit (TaKaRa, Dalian, China). PCR was performed using SYBR Green Premix Ex Taq (TaKaRa) with specific primer pairs (all primer pairs are listed in S1 Table) on an Applied Biosystems Stepone real-time PCR System. The thermocycler program was as follows: 5 min at 95°C; 40 cycles of 30s at 95°C, 45 sec at T_Anneal_, and 30s at 72°C; and 5 min at 72°C. Relative quantification of mRNA was conducted using the comparative 2^−ΔΔCt^ method with GAPDH as the reference gene.

### Western blot analysis

Protein lysates were mixed with β-mercaptoethanol, glycerin, and bromophenol-blue, and allowed to incubate at 95°C for 5 min. Equal amounts of protein from each sample were separated on 10% SDS-polyacrylamide gels and transferred to polyvinylidene fluoride (PVDF) membranes (Millipore, Bedford, MA). Membranes were blocked in 5% nonfat dry milk for 2 h at 4°C, incubated overnight at 4°C with anti-IL-1β (Abcam, Cambridge, MA) and anti-PAK6 (Santa Cruz Biotechnology), followed by reaction with horseradish peroxidase (HRP)-conjugated secondary antibodies (Pierce, Rockford, IL). Membranes were developed with enhanced chemiluminescence reagent (Cell Signaling, Beverly, MA) prior to exposure to Kodak X-Omat Blue Film (NEN life science, Boston, MA). Measurements of band signal intensity were conducted with Grab-it 2.5 and Gelwork software.

### Statistical Analysis

All data are expressed as means ± SEM, and analyzed by means of SPSS 15.0 software (SPSS, Chicago, IL). Differences between groups were tested using Student's t-test and one-way ANOVA.

## Results

### Differentially expressed genes in crushed sciatic nerve

**Fig 1:**
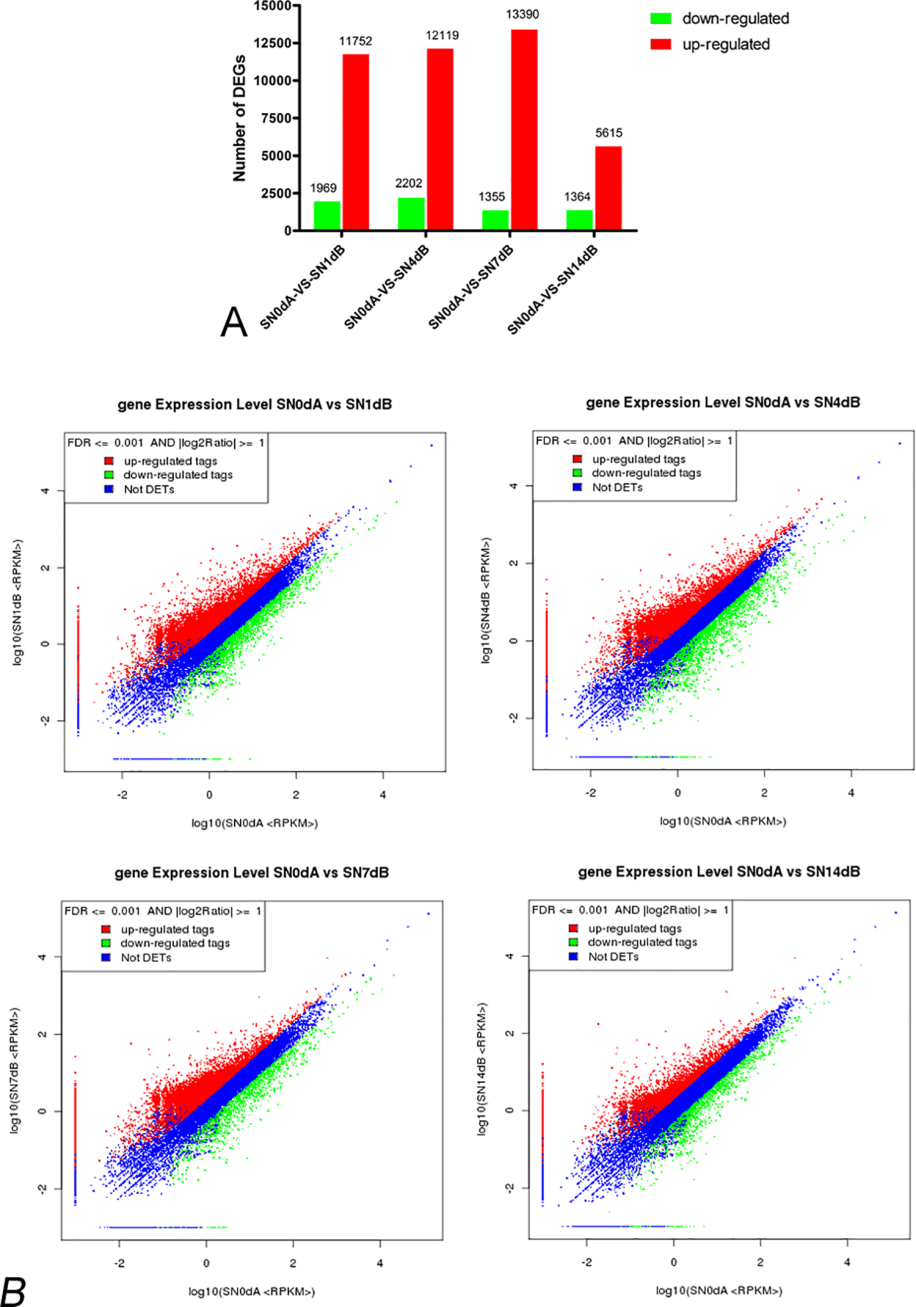
Overview of differentially expressed genes. The (A) bar graph and (B) charts show that a huge number of genes were differentially expressed in injured sciatic nerves at 1, 4, 7, and 14 days post nerve injury compared to those at 0 day post nerve injury with a fold change ≥ 2 (Log_2_Ratio ≥ 1) and a false discover rate (FDR) ≤ 0.001. The value of Log_2_ratio is shown in the X-axis and Y-axis of the chart, where red and green dots indicate up-regulation and down-regulation, respectively, while blue dots indicate no significant difference.

Deep sequencing was applied to determine the gene expression pattern in injured sciatic nerves at different time points. We identified that 35,728, 38,024, 36,857, 37,513, and 36,403 genes were expressed at 0, 1, 4, 7, and 14 days post sciatic nerve crush, respectively. Compared with expressed genes at 0 day post injury, nearly 1/3 of genes were differentially expressed (> 2 fold change, FDR ≤ 0.001) at 1 day post injury, and most of them were up-regulated (11,752 up-regulated genes and 1,969 down-regulated genes); the number of differentially expressed genes was greater at 4 or 7 days post injury than at 1 day post injury, and most of differentially expressed genes at 4 or 7 days post injury were also up-regulated; but a dramatic decrease in the number of differentially expressed genes appeared at 14 days post injury, when 5,615 genes were up-regulated and 1,364 genes were down-regulated (Fig 1). A full list of all differentially expressed genes at different time points is shown in S2 Table.

### Diseases and biological functions

**Fig 2:**
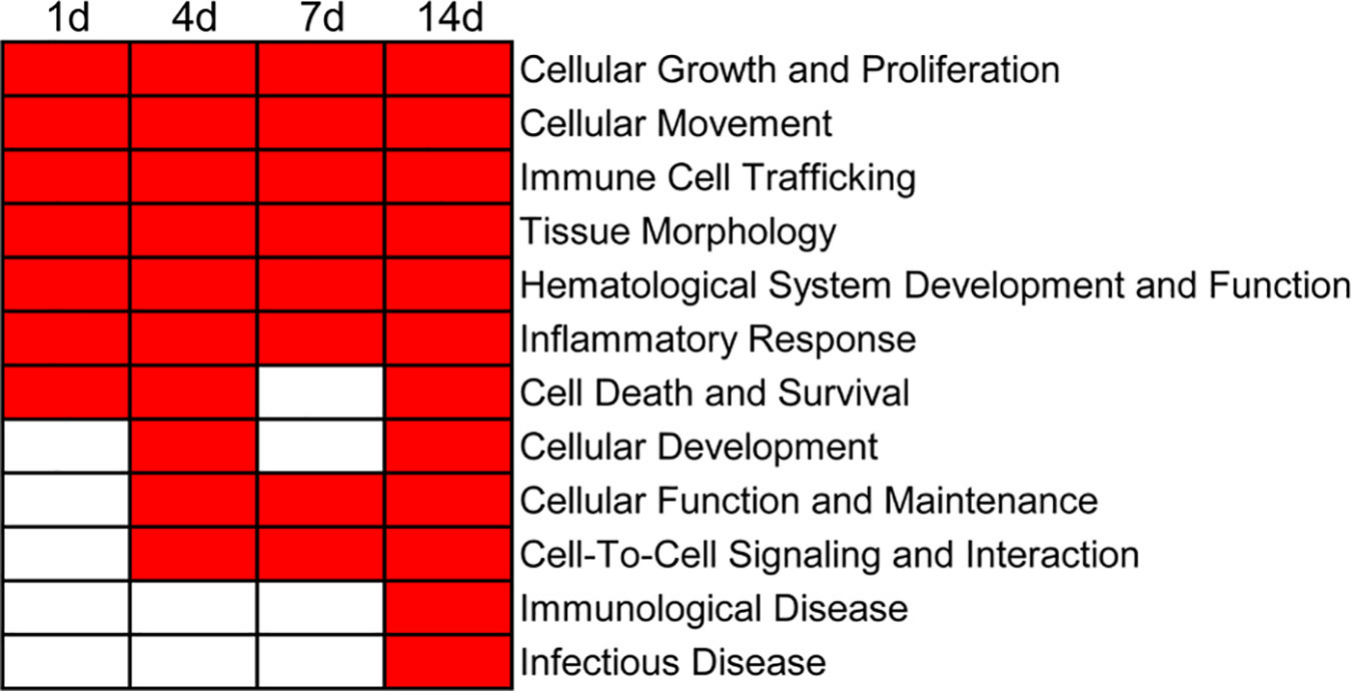
Top enriched diseases and biological functions with a p-value < 10^−50^. Diseases and biological functions with a p-value < 10^−50^ are labeled in red color while diseases and biological functions with a p-value > 10^−50^ are labeled in white color.

A huge number of genes were temporally differentially expressed in the injured sciatic nerve post nerve crush. This finding suggested that peripheral nerve injury induced a dramatic effect on the transcriptome landscape of the injured nerve. To better understand the involvement of biological processes following sciatic nerve crush, differentially expressed genes were correlated to diseases and biological functions using IPA according to IPKB. According to the corresponding p-value (smaller or larger than 10^−50^), the top enriched categories of disease and biological functions at different time points were identified (Fig 2). A full list of diseases and biological functions and involved molecules is shown in S3 Table.

### Canonical pathways analysis

**Fig 3:**
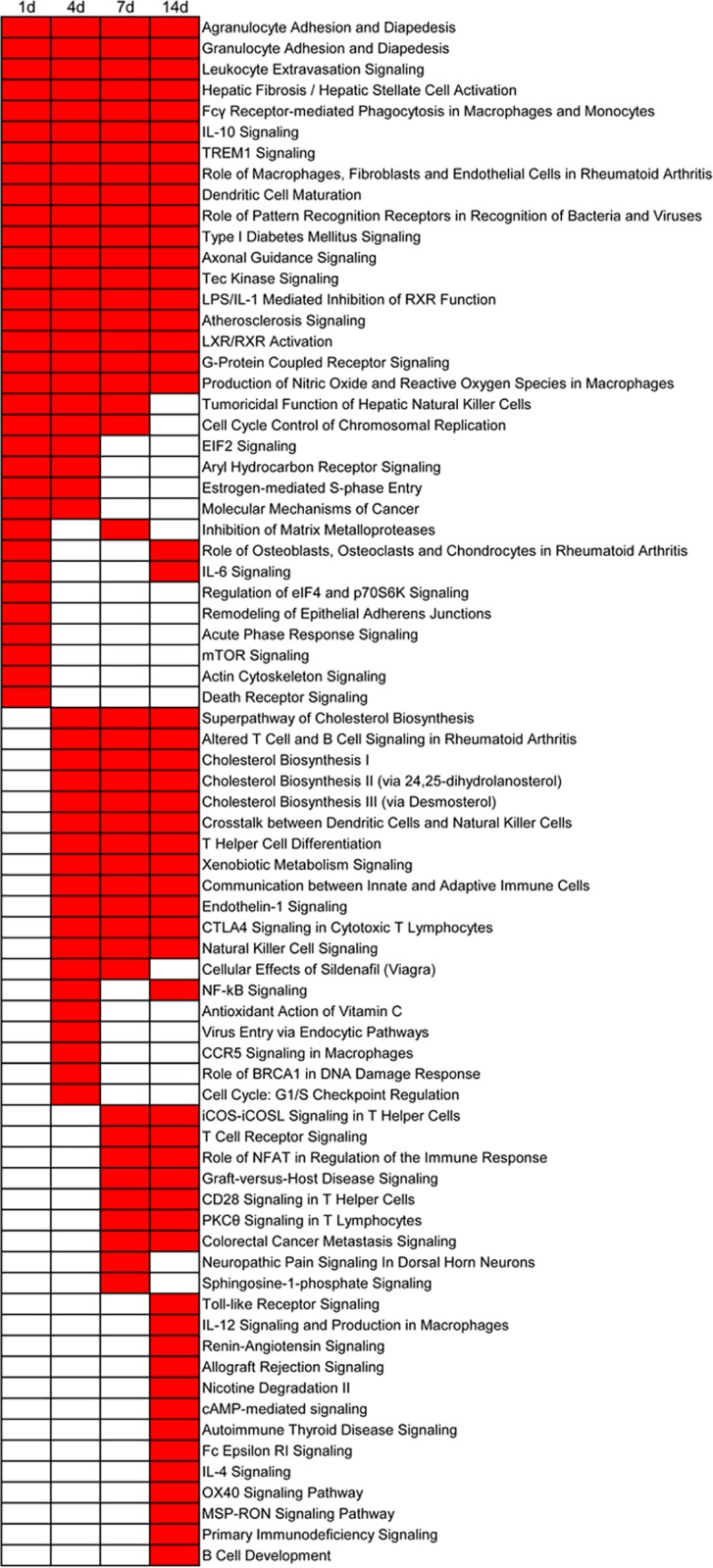
Top enriched canonical pathways with a p-value < 10^−5^. Canonical pathways with a p-value < 10^−5^ are labeled in red color while canonical pathways with a p-value > 10^−5^ are labeled in white color.

Diseases and biological functions provided a preliminary insight into the cellular and molecular regulation of peripheral nerve regeneration. Canonical pathways associated with differentially expressed genes at each time point were also analyzed. The top enriched categories at different time points are displayed with the p-value threshold set at 10^−5^ (Fig 3). A full list of canonical pathways and involved molecules at 1, 4, 7, and 14 days post sciatic nerve crush is provided by S4 Table.

After identifying top enriched canonical pathways, we used z-score, a scoring algorithm, to determine the match between the observed gene expression from transcriptome sequencing and the literature based-gene expression, and then identify key signaling pathways that were more related to biological processes and functions. A z-score > 2 (consistent with original prediction) or < –2 (opposite to original prediction) was considered as meaningful. Afterwards, several key canonical signaling pathways with a high solute z-score, including acute phase response signaling, TREM1 signaling, LXR/RXR activation, and axonal guidance signaling. were chosen for further investigation. To facilitate temporal analysis, the post-injury period was artificially divided into 3 distinct phases: acute, sub-acute, and post-acute stages, which corresponded to 1 day, 4 and 7 days, and 14 days post injury, respectively.

### Acute phase response signaling

**Fig 4:**
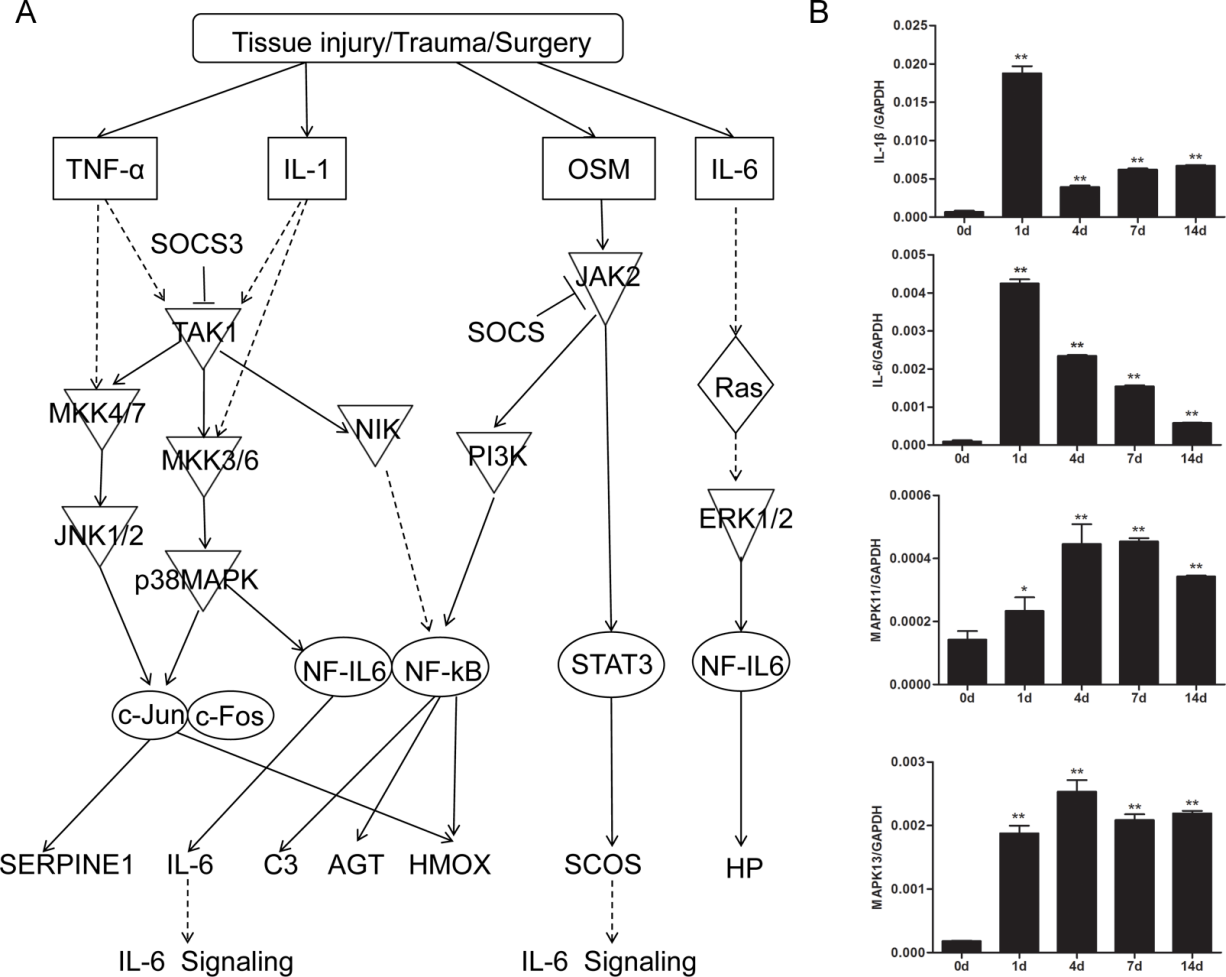
Acute phase response signaling was strongly activated at the early acute stage post nerve injury and kept activated during the whole post-injury period. (A) A schematic network of acute phase response signaling. (B) qPCR determination for mRNA expressions of IL-1β, IL-6, MAPK11, and MAPK13 at 0, 1, 4, 7, and 14 days post injury. The relative level is normalized to GAPDH. Summarized data are from 3 independent experiments. Values are shown as mean ± SEM. The asterisk indicates significant difference (*, P < 0.05; **, P < 0.01).

Acute phase response is a systemic reaction of the organism triggered by tissue injury, trauma, neoplastic growth, or immunological disorders [19]. Following sciatic nerve crush, a series of events occurs at the injury site, including the secretion of pro-inflammatory cytokines and inflammatory mediators, the activation of inflammatory cells, and the increase in plasma concentrations of the positive acute-phase proteins. At the acute stage (1 day post injury) following nerve injury, pro-inflammatory cytokines, such as tumor necrosis factor-α (TNF-α), interleukin-1 (IL-1), oncostatin M (OSM), interleukin-6 (IL-6), were up-regulated. Through the activation of p38 MAPK, JNK, STAT3, and/or PI3K signaling pathways, the plasma concentrations of positive acute-phase proteins were increased and the plasma concentrations of negative acute-phase proteins were decreased, leading to a rapid inflammatory response (Fig 4A and S3 Fig). However, the use of 0 day group as a general sham-surgery control seemed not to eliminate the influences of stress response and inflammatory reaction on the gene expression data, and the elevated inflammatory response following sciatic nerve crush might partly be induced by the surgery procedure.

According to IPA analysis, the acute phase response signaling was ranked the 26^th^ place at day 1 post nerve injury with a p-value of 10^−5.6^ and a z-score of 4.901, affecting 62 genes that are differentially expressed according to transcriptome sequencing. The affected molecules include IL-1, IL-6, OSM, etc. A full list of differentially expressed gene is shown in S5 Table. Starting from 4 days post injury, a relatively smaller number of differentially expressed genes were involved in acute phase response signaling pathway: 53, 40, and 39 genes were involved at 4, 7, and 14 days post injury, respectively. The decreases in significance (increased p-value), z-score, and the number of involved differentially expressed genes suggested that acute phase response was mainly observed at the acute stage (1 day) post injury.

The expression patterns of several key molecules involved in acute phase signaling were validated using qPCR. Consistent with transcriptome sequencing, qPCR showed that IL-1β and IL-6, two cytokines that activate downstream kinases (p38 MAPK and PI3K) and mediate inflammatory responses, were highly expressed at 1 day post injury. Later on, the expression level of IL-1β or IL-6 was decreased, but still higher than that at 0 day post injury. The mRNA expression of MAPK11 (p38 MAPK-β) or MAPK13 (p38 MAPK-δ) was increased starting from 1 up to 14 days post injury, and peaked at 4 days post injury, as compared to that at 0 day post injury (Fig 4B). Moreover, outcome from Western blot further suggested that the expression level of IL-1β were kept up-regulated following nerve injury (S4A Fig).

Taken together, our data suggested that the acute phase inflammatory response initiated immediately after peripheral nerve injury (at an acute stage).

### TREM1 signaling

**Fig 5:**
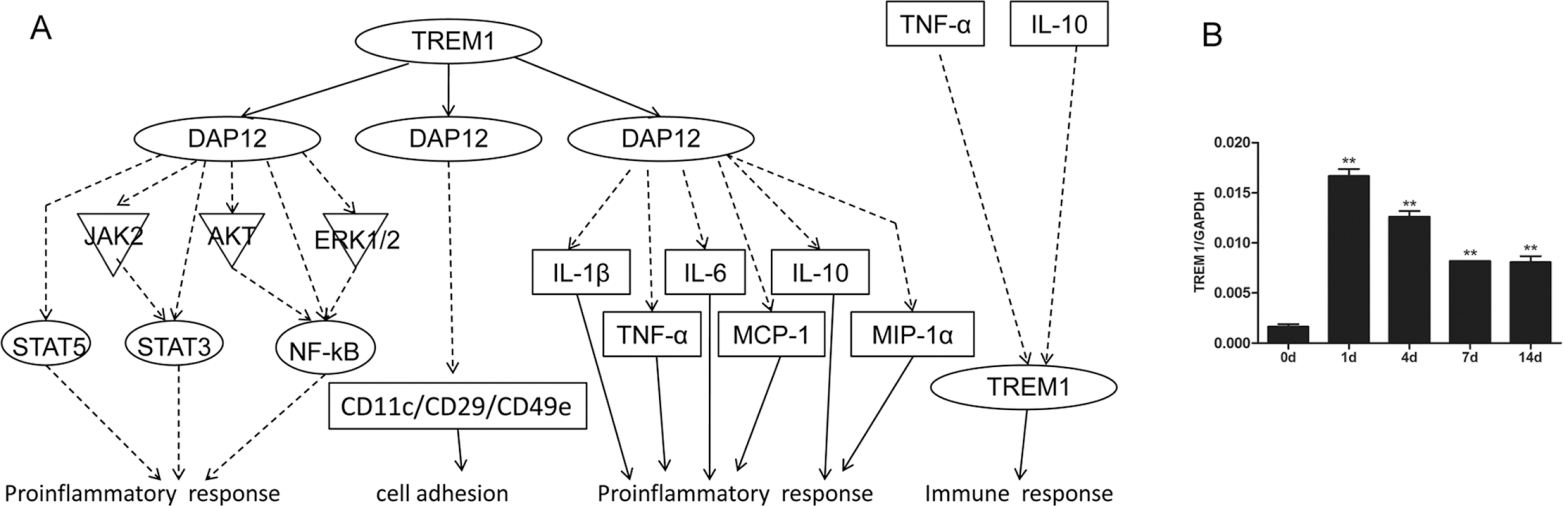
TREM1 signaling was kept activated following nerve injury. (A) The schematic network of TREM1 signaling. (B) Expression of mRNA coding TREM1 at 0, 1, 4, 7, and 14 days. The expression of TREM1 is referenced to GAPDH. Summarized data are from 3 independent experiments. Values are shown as mean ± SEM. The asterisk indicates significant difference (**, P < 0.01).

TREM1 signaling is another inflammation and immune response-related signaling pathway, which was ranked higher over the time span we examined. The activation of TREM1 triggers several signaling pathways, such as JAK2 and AKT, phosphorylates signal transducer of activation of transcription (STAT3 and STAT5) and NF-κB, and elevates the expression of the inflammatory response-related genes. The activation of TREM1 also triggers the expression and activation of cytokines and chemokines, such as IL-1β, IL-6, IL-10, TNF-α, and monocyte chemotactic protein 1 (MCP-1), and macrophage inflammatory protein-1α (MIP-1α), which in turn regulates the expression of TREM1. Moreover, TREM1 up-regulates the cell surface proteins and promotes cell adhesion (Fig 5A and S5 Fig).

Among all differentially expressed genes involved in TREM1 signaling, as listed in S6 Table, TREM1 was chosen for validation of the mRNA expression level by qPCR. Similar as IL-1β and IL-6, TREM1 was highly expressed at 1, 4, 7, and 14 days post injury as compared to that at 0 day post injury, suggesting that TREM1 signaling was critical in all 3 stages of post-injury period (Fig 5B).

### LXR/RXR activation

**Fig 6:**
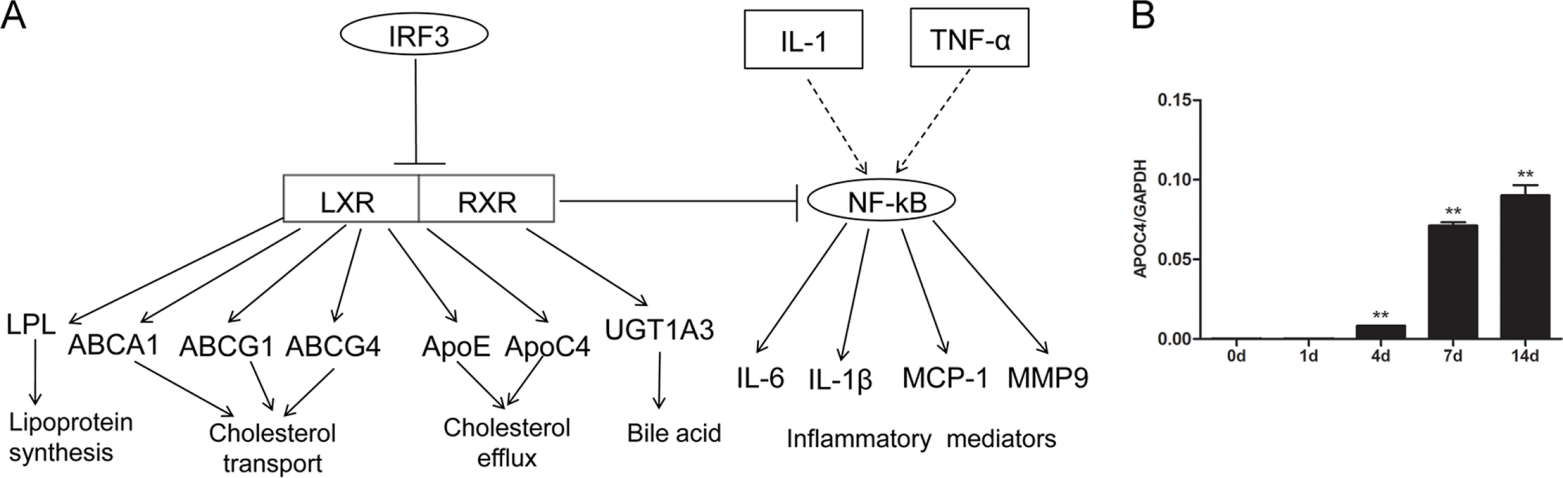
LXR/RXR activation was stepwise activated during nerve regeneration. (A) The schematic network of LXR/RXR activation signaling. (B) Expression of mRNA coding APOC4 at 0, 1, 4, 7, and 14 days. The expression of APOC4 is referenced to GAPDH. Summarized data are from 3 independent experiments. Values are shown as mean ± SEM. The asterisk indicates significant difference (**, P < 0.01).

The activation of LXR/RXR affects both immune response and lipid metabolism (Fig 6A and S6 Fig). As mentioned above, lots of genes that belong to the LXR/RXR activation canonical pathway were differentially expressed (S7 Table) and the importance of LXR/RXR signaling might increase with time, implying that LXR/RXR contributes to nerve regeneration through affecting lipid metabolism and molecular transport.

In agreement to transcriptome sequencing, qPCR data demonstrated a robust increase in the mRNA expression of apolipoprotein C-IV (APOC4), a lipid-binding protein involved in the transport of lipids and other small hydrophobic molecules in lipid metabolism, at 4, 7, and 14 days post injury (Fig 6B).

### Axonal guidance signaling

**Fig 7:**
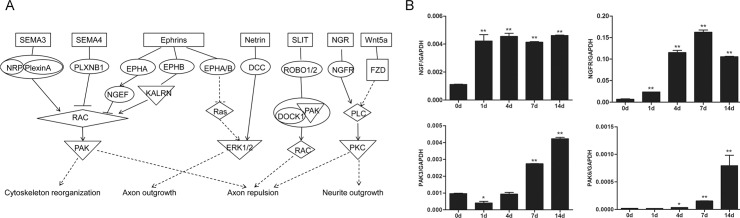
Axonal guidance signaling was activated during nerve repair and regeneration. (A) The schematic network of axonal guidance signaling. (B) Expression of mRNA coding NGF, NGFR, PAK3, and PAK6 at 0, 1, 4, 7, and 14 days. The expressions of target genes are referenced to GAPDH. Summarized data are from 3 independent experiments. Values are shown as mean ± SEM. The asterisk indicates significant difference (*, P < 0.05; **, P < 0.01).

Axonal guidance signaling related to the nerve regeneration process was further studied. Following nerve injury, cytoskeleton started reorganization and axons started outgrowth through the regulation of attractive and repulsive guidance cues, including netrins, slits, semaphorins, ephrins and their receptors (Fig 7A and S7 Fig).

Timely regrowth of axons and the directional extension and migration to their synaptic targets are the basis of the functional recovery of injured nerves. The axonal guidance signaling kept playing a significant role in nerve regeneration, with a p-value of 10^−7^, 10^−6.68^, 10^−6.61^ and 10^−5.16^ at 1, 4, 7, and 14 days post injury, respectively, and 138, 135, 109, and 80 differentially expressed genes were involved in the axonal guidance signaling pathway at 1, 4, 7, and 14 days post injury, respectively (S8 Table).

The early detection of axonal guidance signaling seems to conflict with the existing knowledge that the process of axon regrowth and remyelination do not happen in the acute stage post nerve injury. A detailed study indicated that some molecules involved in the axonal guidance signaling pathway, such as nerve growth factor (NGF) and nerve growth factor receptor (NGFR) were kept up-regulated, while other molecules, such as p21-activated kinases: PAK3 and PAK6, were progressively up-regulated.

NGF is a key growth factor of neuron growth and survival that contributes to the development and phenotype maintenance of the PNS. Consistent with transcriptome sequencing results, notably up-regulated mRNA expressions of NGF and NGFR were observed (Fig 7B). Meanwhile, a previous study in our group suggested that not only the gene expression, but also the protein expression of NGF were significantly increased following sciatic nerve injury [20], providing further verification from the protein aspect.

PAK3 and PAK6 are critical molecules of cytoskeleton reorganization, dendrite spine morphogenesis, and synapse formation and plasticity. The mRNA levels of PAK3 and PAK6, as determined by qPCR (Fig 7B), were in accordance with those by deep RNA sequencing. Data from Western blots suggested that protein expression levels of PAK6 were almost undectable at 0, 1, 4, and 7 days following nerve injury but reached a detectable level at 14 days (S4B Fig). The significant increased protein expression of PAK6 was consistent with qRCR and deep sequencing results.

Taken together, these outcomes suggested that axonal guidance was stimulated early on and axonal regrowth was later triggered after peripheral nerve injury.

## Discussion

**Fig 8:**
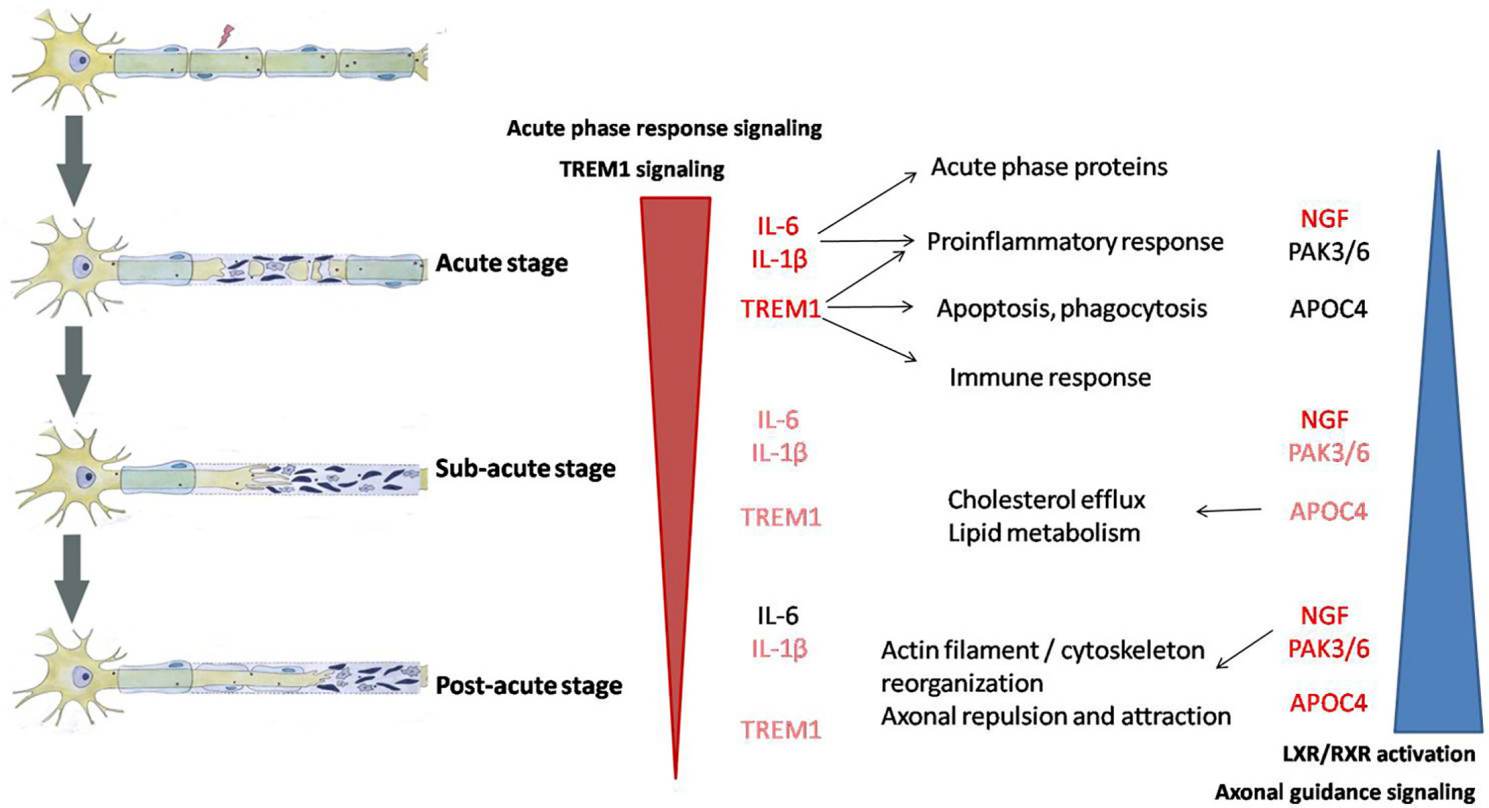
Schematic diagram showing 3 distinct phases within the post-injury period of 14 days and the involved key biological functions in each phase.

A rat model of sciatic nerve crush is commonly used for peripheral nerve regeneration studies. Following sciatic nerve crush, nerve function is immediately disrupted, while nerve regeneration and functional recovery occur at two weeks post injury [21]. In the current study, we examined the molecular changes in the injured sciatic nerve at 1, 4, 7, and 14 days post injury. A huge number of differentially expressed genes has been identified by deep sequencing. Using IPA analysis, we gained a comprehensive view of molecular changes and the related functions and pathways. According to the temporally differential expression patterns of a large number of genes, 3 distinct phases were defined within the post-injury period of 14 days: the acute (1 day), sub-acute (4 and 4 days), and post-acute (14 days) stages (Fig 8). Each stage showed its own characteristics of gene expression, which were associated with different categories of diseases and biological functions and canonical pathways.

One of the top enriched categories of diseases and biological functions at the acute stage was cellular growth and proliferation, suggesting that instant cell survival and compensatory cell growth was critical for neuronal regrowth and nerve regeneration. The top enriched functional categories also include cellular movement and tissue morphology. As is well known, cell movement and migration, together with cell growth, are vital for organ development and post-developmental tissue formation. Previous studies have shown that enhanced Schwann cell migration would promote nerve regeneration [22, 23]. The results in the current study suggest that at the acute stage, cells started response to stimuli induced by sciatic nerve crush and migrated toward external signals for encouraging wound healing and regeneration of injured nerves. Inflammatory and immune response-related functions, such as immune cell trafficking and inflammatory response, were also top enriched categories in that inflammatory response and inflammatory cytokines facilitate the recruitment of immune cells (e.g. macrophages and monocytes) [24, 25], which promote nerve regeneration by cleaning the axonal remnants and myelin debris, secreting growth factors, and regulating the composition of the extracellular matrix [26–29]. Accordingly, elevated inflammatory and immune response and elevated trafficking of immune cells, as observed in the current study, would advance the recruitment of immune cells, the clearance of debris, and axonal regeneration.

Top enriched categories at the sub-acute stage include cellular growth and proliferation, tissue morphology and cellular movement, suggesting that cell growth and movement were still important at this stage. Noteworthy categories, such as cellular function and maintenance and cell-to-cell signaling and maintenance were enriched, which might be because of more cells switching from the growth state to the movement and functioning state for rebuilding the injured nerve.

Within the sub-acute stage, biological functions involved at 4 and 7 days post injury were roughly comparable to each other. It is noticeable that the category of hematological system development and function was listed in the first place at 7 days post injury (Fig 2 and S3 Table). Angiogenesis is important for nerve regeneration as the newly formed blood vessels supply oxygen and nutrients, co-align with regenerated nerves, and promote nerve regeneration and functional recovery [30–32]. In contrast, the category hematological system development and function is listed in the 6^th^ and 3^rd^ place at 1 and 4 days post injury, respectively demonstrating that genes involved in blood vessel formation were more regulated at the sub-acute stage than at the acute stage.

At the post-acute stage, a relatively smaller number of differentially expressed genes were identified (Fig 1), and biological functions related to tissue formation, such as cellular movement, tissue morphology, hematological system development and function, and cellular function and maintenance, were enriched, accompanied by the onset of nerve regeneration.

Using the IPA analysis, we identified that top enriched canonical pathways at the acute phase were the categories of cellular immune responses, cellular growth and proliferation, cellular apoptosis, cellular death, cell cycle regulation, cellular movement, cell-cell signaling, lipid metabolism and molecular transport, organismal growth and development, and disease-specific pathways. These top canonical pathways provided a conclusion consistent with that drawn from analysis of top diseases and biological functions. The consistent conclusions were that cellular growth and death, cellular movement, and immune response were critical at the acute stage.

Just like diseases and biological functions, canonical pathways at 4 and 7 days post injury were similar to each other. A distinguishing feature at this sub-acute stage was the appearance of lipid biosynthesis-related canonical pathways, such as superpathway of cholesterol biosynthesis, cholesterol biosynthesis I, cholesterol biosynthesis II (via 24, 25-dihydrolanosterol), and cholesterol biosynthesis III (via desmosterol). The synthesis and intracellular movement of membrane lipids are required for axonal elongation and remyelination during nerve regeneration [33, 34]. Another top enriched canonical signaling pathway was natural killer cell signaling. The activation of natural killer (NK) cells may trigger cytolytic programs and stimulate the secretions of cytokines and/or chemokines, which are essential for nerve regeneration [35]. NK cells increase the expression of TrkA NGFR [36]. A previous study focusing on liver regeneration demonstrates that the depletion of NK cells and NK T cells leads to impaired liver regeneration after partial hepatectomy [37]. Therefore, elevated NK cell signaling, as observed at the sub-acute stage, might be beneficial to peripheral nerve regeneration.

At the post-acute stage, one of the most remarkable canonical pathways was LXR/RXR activation. The liver X receptor (LXR) forms heterodimer with retinoic acid receptor (RXR), and activated LXR/RXR regulates inflammation as well as the homeostasis of cholesterol and fatty acid. The LXR/RXR activation pathway were detected at all time points following nerve crush, listed in the 20th, 16th, 10th, and 4th place with a p-value of 10^−5.97^, 10^−7.53^, 10^−8.77^, and 10^−13^ at 1, 4, 7, and 14 days post injury, respectively. This result implied that this canonical pathway went through the whole process of nerve regeneration.

Considering a massive number of molecules that were involved in biological functions and canonical pathways, we chose key molecules for validation of their mRNA and protein expression by qPCR and Western blot analysis respectively. In this way, some interesting results were obtained as follows.

At the acute stage, acute phase response and TREM1 signaling were instantly activated, and inflammation and immune response were immediately triggered. At the sub-acute stage, inflammation and immune response were slightly less important while cellular movement and tissue morphology became more important as compared to at the acute stage. At the post-acute stage, the number of differentially expressed genes reduced to ~7000, which was significantly less than the number of differentially expressed genes in the acute or sub-acute stage. This comparison implied a certain level of recovery occurring at this stage. Activation of LXR/RXR signaling and axonal guidance signaling also suggested that in this stage, such biological processes as lipid metabolism, actin filament/cytoskeleton reorganization, and axonal guidance might contribute to nerve regeneration.

Inflammatory reaction is ubiquitously and rapidly elicited following trauma injury to the central and peripheral nervous system [38, 39]. During Wallerian degeneration after peripheral nerve injury, pro-inflammatory cytokines, such as TNF-α, IL-1, and IL-6, are produced in the distal stump and/or recruited through circulation. The up-regulated cytokines activate macrophages to clean axonal and myelin debris and stimulate subsequent axonal regrowth [24, 39]. At the later stage of peripheral nerve injury, the expression levels of anti-inflammatory cytokines are up-regulated to attenuate the inflammatory process [24]. Moreover, different from the central nervous system, where most recruited macrophages are pro-inflammatory, in the peripheral nerve system, some recruited macrophages are anti-inflammatory [40]. Those anti-inflammatory macrophages may help suppressing inflammation and promoting nerve regeneration [41]. The results of the current study also showed that some inflammatory response-related genes were differentially expressed following sciatic nerve crush. To better clarify the involvement of inflammatory responses in the acute stage, the paired sham control needs to be used to analyze the changes and effects of inflammatory response-related genes in future studies. Canonical signaling pathways analysis also showed that the acute phase response signaling was vital at the early acute stage while the TREM1 signaling was vital during the whole process of nerve regeneration because some anti-inflammatory cytokines (e.g. IL-10) were involved in TREM1 signaling. Further study will be performed to determine the expression levels and the specific roles of pro-inflammatory and anti-inflammation cytokines as well as pro-inflammatory and anti-inflammatory macrophages at each time point, aiming to get a better understanding of the correlation between inflammatory response and peripheral nerve regeneration.

Cellular movement and morphology is another noticeable biological process. Chemotaxis, the cellular movement in response to stimulus, promotes migration of immune cells to the injury site and thus affects Wallerian degeneration. Additionally, cellular movement also contributes to wound healing and the development of newly-formed tissue. Active migration of Schwann cells promotes axon outgrowth and elongation [12]. Our results from deep sequencing suggested that cellular movement was critical at the acute stage post injury and became increasingly important during nerve regeneration. Collectively, cellular development, migration, and morphology may be the key biological elements during axon elongation and nerve regeneration.

Lipid metabolism and transport was also remarkable especially at the post-acute stage. The activation of LXR/RXR signaling pathway was identified as one of the top enriched canonical signaling pathways in mice after spinal cord injury [42]. The activation of LXR/RXR in both central nerve injury and peripheral nerve injury may be associated with the indispensability of LXR/RXR during nerve regeneration. Previous studies have verified the importance of synthesis and transport of lipids (especially lipoproteins and apoliproproteins) for nerve regeneration [34, 43, 44]. In the current study, bioinformatic analysis indicated the importance of other molecules in the LXR/RXR activation signaling pathway. A subsequent investigation of ATP-binding cassette transporters, lipoproteins, apoliproproteins, and related lipase may provide a clue to the linkage of lipid metabolism and transport with nerve regeneration.

In conclusion, we generated an integrated global view of gene expression patterns during peripheral nerve injury and regeneration by using deep RNA sequencing. IPA analysis further enabled us to identify key biological functions and canonical pathways. Overall, our results may help to elucidate the molecular mechanisms underlying peripheral nerve regeneration, and to identify potential therapeutic targets for the treatment of peripheral nerve injury.

## Supporting Information

S1 Fig Schematic depiction of deep sequencing and bioinformatics analysis. After the total RNA extraction and DNAase I treatment, mRNA was isolated and then fragmented into short fragments. Based on the short mRNA fragments, cDNA was synthesized, went through size selection and PCR amplification, and sequenced. Produced raw reads were filtered into clean reads and those clean reads were aligned to the reference sequences. Downstream analysis was performed on alignments results that passed quality control.
(TIF)

S2 Fig Quality control of sequencing data. The quality of sequencing data was tested based on (A) base composition analysis of raw reads. Position 1–90 bp on the X axis represents read 1 while position 91–180 bp represents read 2. Curves represent the base percentage compositions of adenine (A), thymine (T), guanine (G), and cytosine (C) along reads. (B) quality distribution of bases along reads. Each dot represents the quality value of the corresponding position along reads with a quality value less than 20 is considered as low. (C) randomness assessment. The curve presents the distributions of number of reads along the relative position in genes.
(TIF)

S3 Fig Schematic network of acute phase response signaling at 0, 1, 4, 7, and 14 days. Differentially expressed genes are marked in color with red indicates up-regulation and green indicates down-regulation. The darker the color, the higher the fold change.
(TIF)

S4 Fig Protein expressions of (A) IL-1β and (B) PAK6 at 0, 1, 4, 7, and 14 days following sciatic nerve crush. Densitometric analysis was conducted to determine protein abundance. The relative abundance of IL-1β or PAK6 is normalized to GAPDH. Results shown are representative of 3 paired observations. Values are shown as mean ± SEM. The asterisk indicates significant difference (*, P < 0.05; **, P < 0.01).
(TIF)

S5 Fig Schematic network of TREM1 signaling at 0, 1, 4, 7, and 14 days. (TIF)

S6 Fig Schematic network of LXR/RXR activation signaling at 0, 1, 4, 7, and 14 days. (TIF)

S7 Fig Schematic network of axonal guidance signaling at 0, 1, 4, 7, and 14 days. (TIF)

S1 Table List of primer pairs for qPCR. (XLSX)

S2 Table List of all differentially expressed genes at 1, 4, 7, and 14 days post sciatic nerve crush. (XLS)

S3 Table List of all categories of diseases and biological functions and involved molecules at 1, 4, 7, and 14 days post sciatic nerve crush. (XLSX)

S4 Table List of all canonical pathways and involved molecules at 1, 4, 7, and 14 days post sciatic nerve crush. (XLSX)

S5 Table List of differentially expressed genes in the acute phase response signaling pathway at 1, 4, 7, and 14 days post sciatic nerve crush. (XLSX)

S6 Table List of differentially expressed genes in the TREM1 signaling pathway at 1, 4, 7, and 14 days post sciatic nerve crush. (XLSX)

S7 Table List of differentially expressed genes in the LXR/RXR activation pathway at 1, 4, 7, and 14 days post sciatic nerve crush. (XLSX)

S8 Table List of differentially expressed genes in the axonal guidance signaling pathway at 1, 4, 7, and 14 days post sciatic nerve crush. (XLSX)
