# Supplementary figures and images for: Deep Sequencing and Bioinformatic Analysis of Lesioned Sciatic Nerves after Crush Injury
Source: PLoS One. 2015 Dec 2;10(12):e0143491. doi: 10.1371/journal.pone.0143491 (PMC4668002; doi:10.1371/journal.pone.0143491)

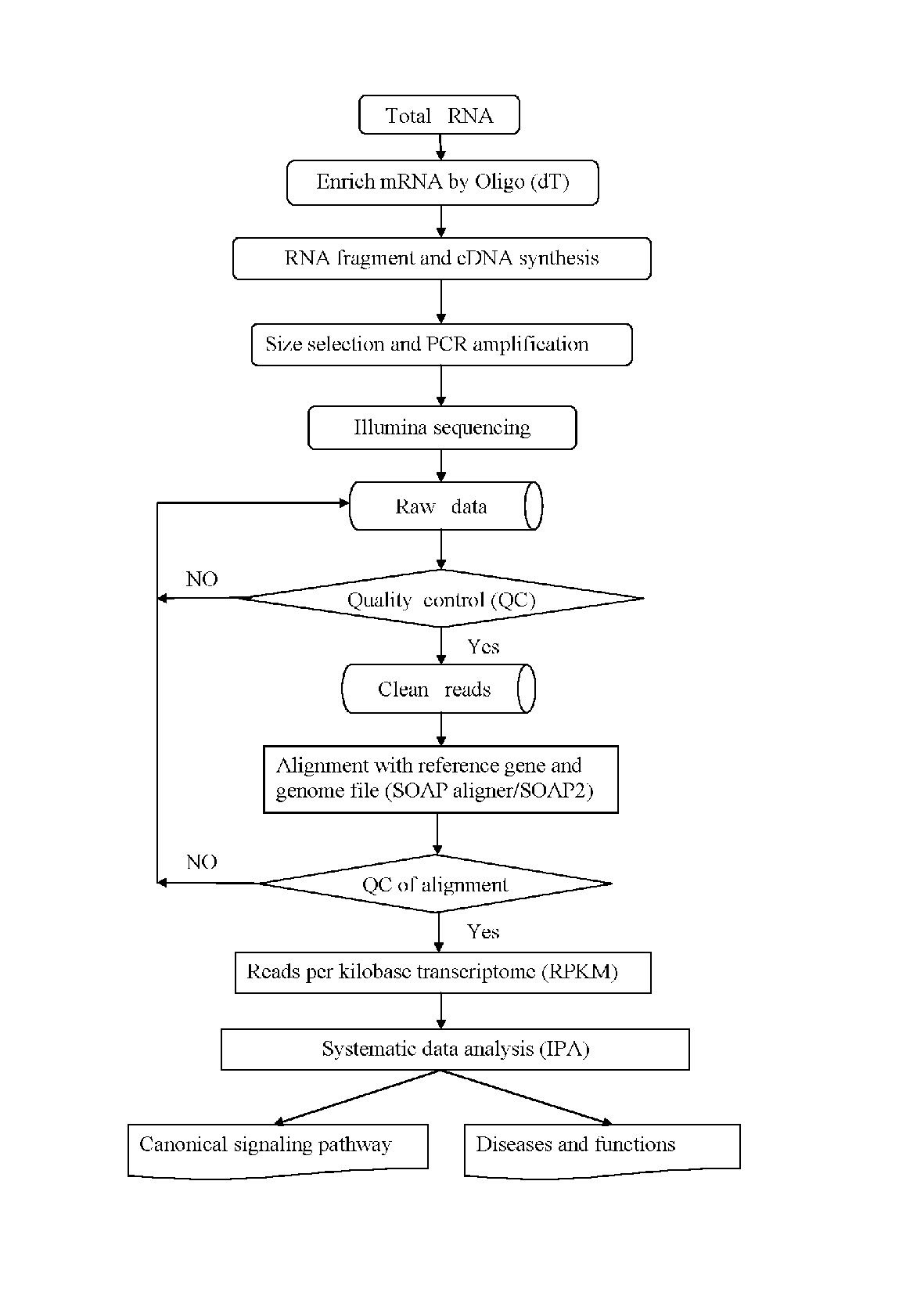

Supplement: S1 Fig — After the total RNA extraction and DNAase I treatment, mRNA was isolated and then fragmented into short fragments. Based on the short mRNA fragments, cDNA was synthesized, went through size selection and PCR amplification, and sequenced. Produced raw reads were filtered into clean reads and those clean reads were aligned to the reference sequences. Downstream analysis was performed on alignments results that passed quality control. (TIF) [file pone.0143491.s001.tif]

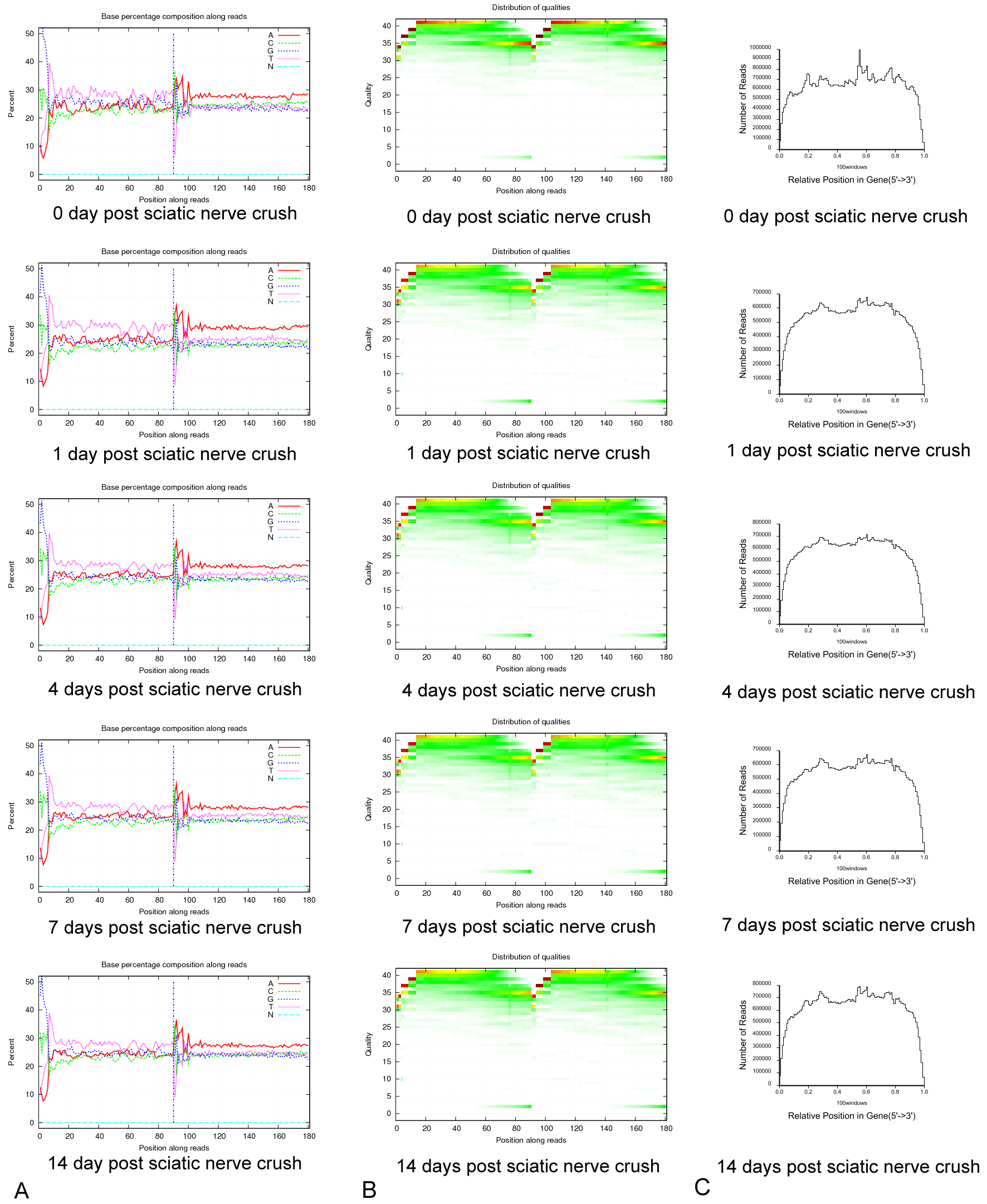

Supplement: S2 Fig — The quality of sequencing data was tested based on (A) base composition analysis of raw reads. Position 1–90 bp on the X axis represents read 1 while position 91–180 bp represents read 2. Curves represent the base percentage compositions of adenine (A), thymine (T), guanine (G), and cytosine (C) along reads. (B) quality distribution of bases along reads. Each dot represents the quality value of the corresponding position along reads with a quality value less than 20 is considered as low. (C) randomness assessment. The curve presents the distributions of number of reads along the relative position in genes. (TIF) [file pone.0143491.s002.tif]

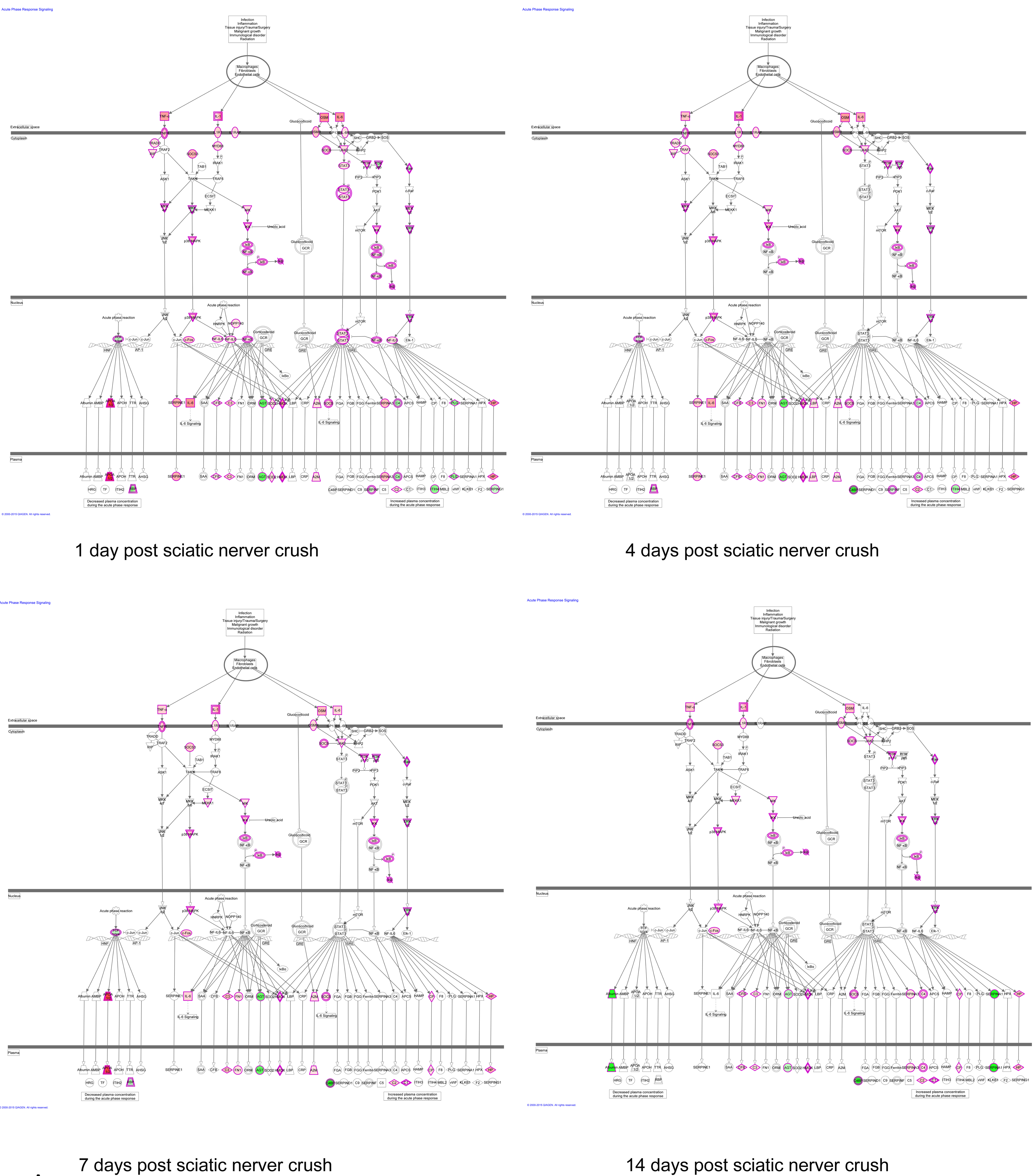

Supplement: S3 Fig — Differentially expressed genes are marked in color with red indicates up-regulation and green indicates down-regulation. The darker the color, the higher the fold change. (TIF) [file pone.0143491.s003.tif]

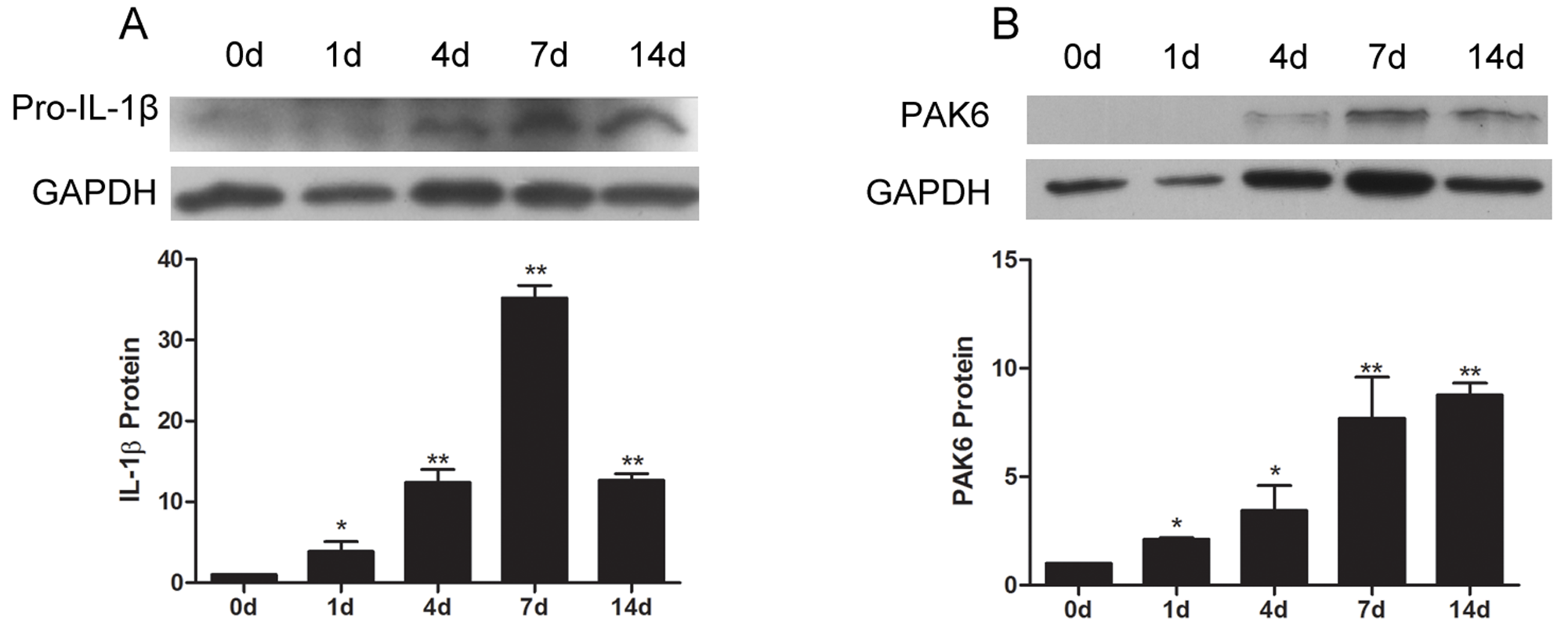

Supplement: S4 Fig — Densitometric analysis was conducted to determine protein abundance. The relative abundance of IL-1β or PAK6 is normalized to GAPDH. Results shown are representative of 3 paired observations. Values are shown as mean ± SEM. The asterisk indicates significant difference (*, P < 0.05; **, P < 0.01). (TIF) [file pone.0143491.s004.tif]

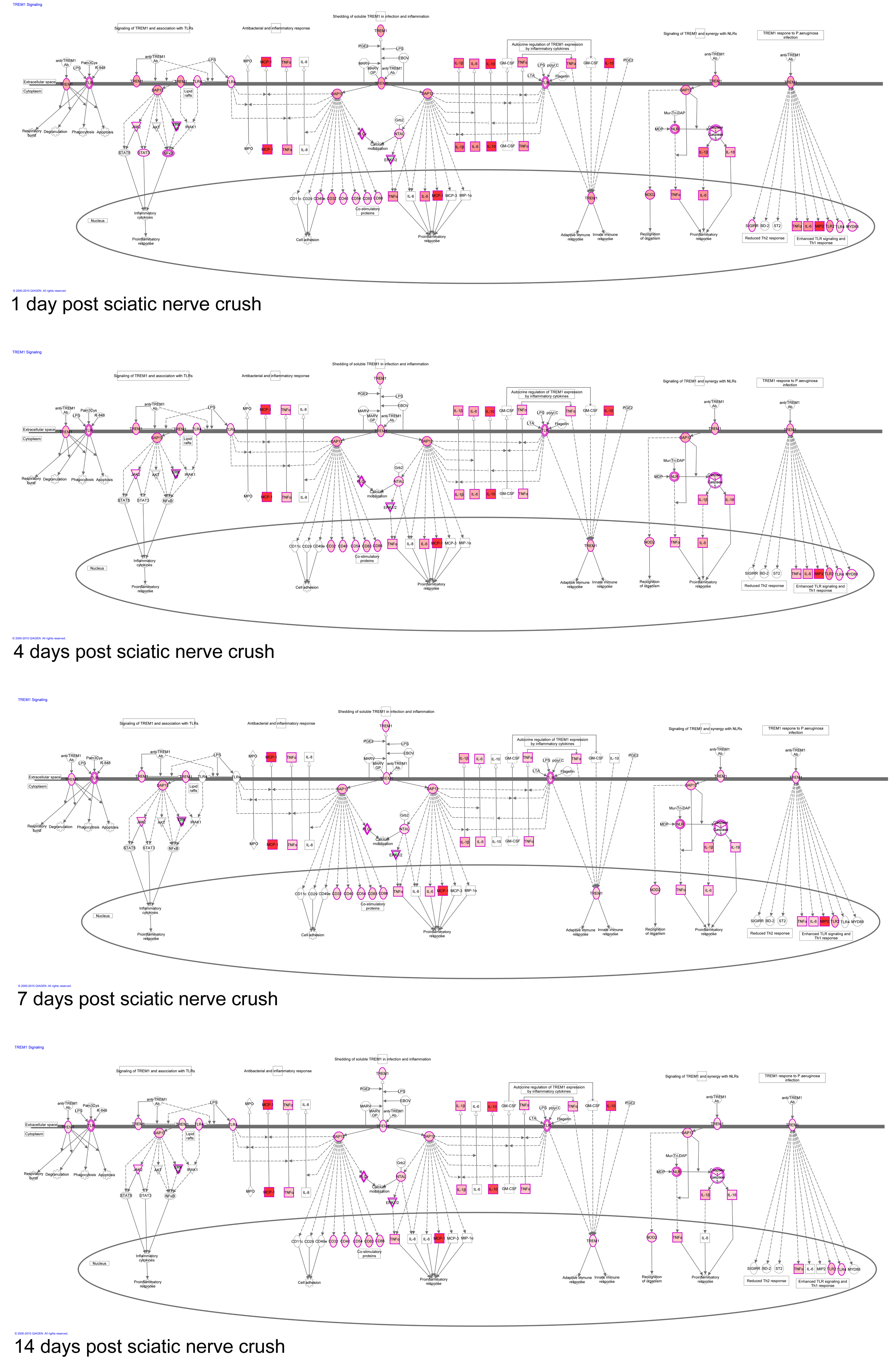

Supplement: S5 Fig — (TIF) [file pone.0143491.s005.tif]

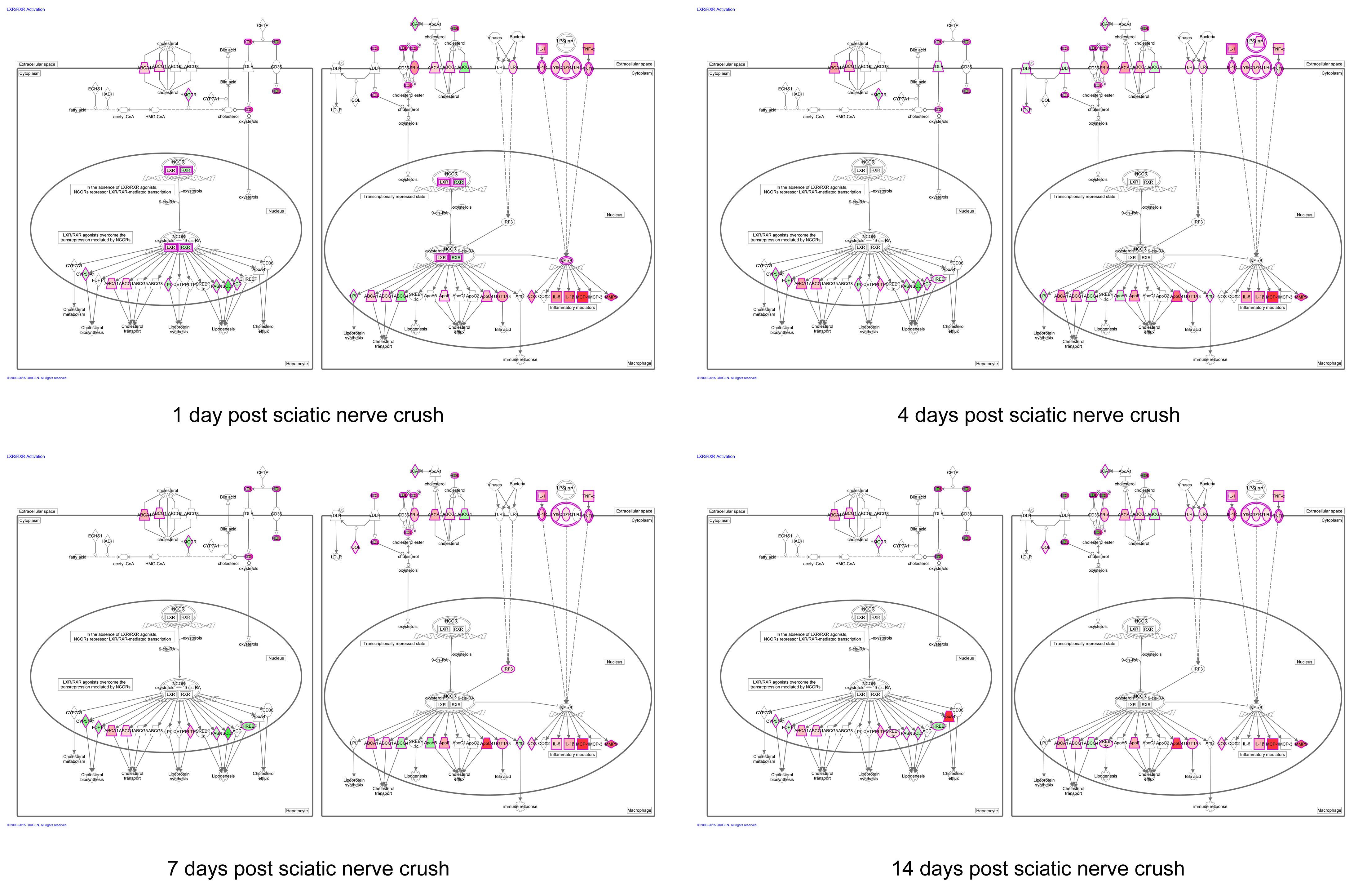

Supplement: S6 Fig — (TIF) [file pone.0143491.s006.tif]

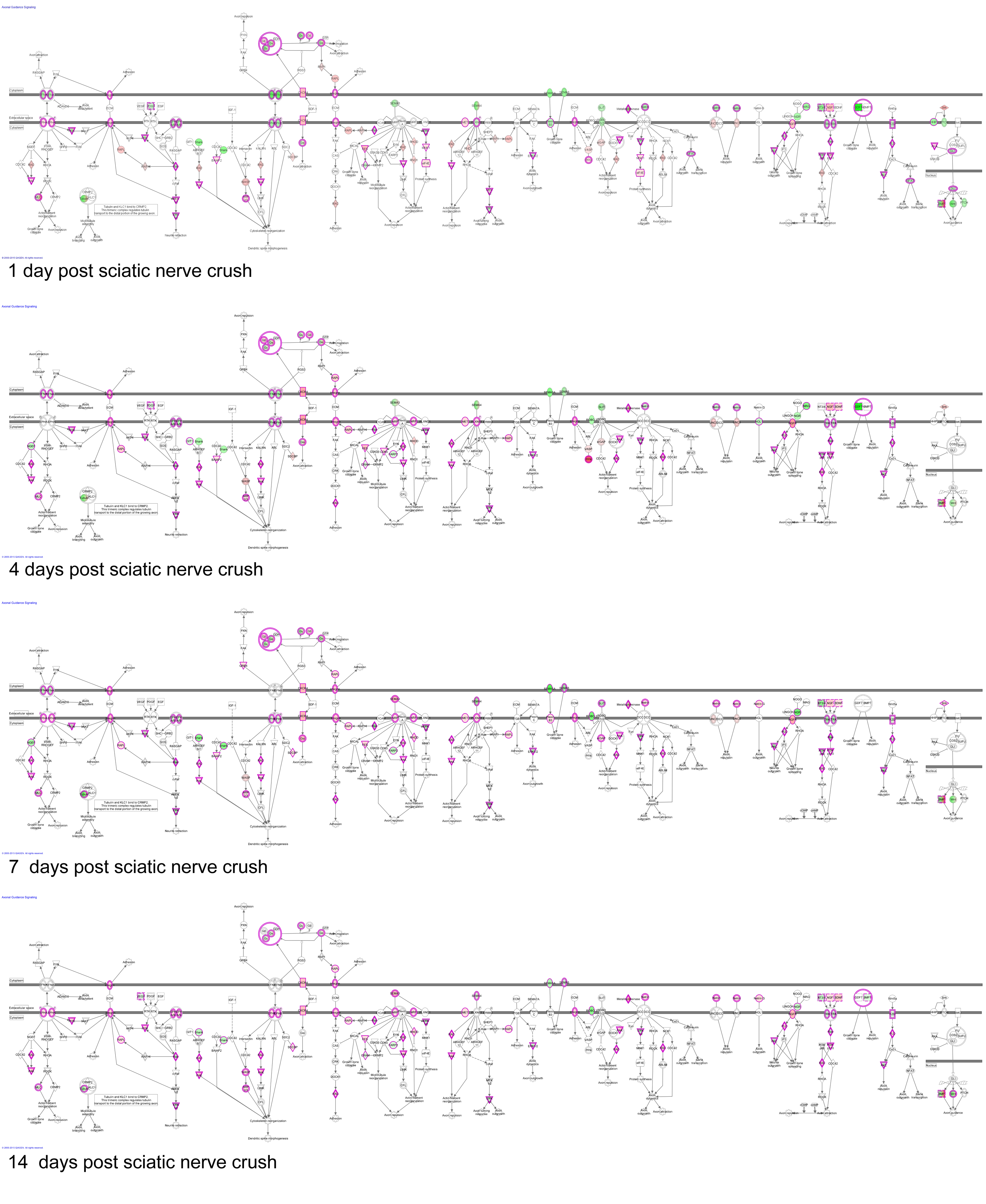

Supplement: S7 Fig — (TIF) [file pone.0143491.s007.tif]
